# The Role of Cyclic Nucleotides in Pituitary Lactotroph Functions

**DOI:** 10.3389/fendo.2013.00122

**Published:** 2013-09-13

**Authors:** Marek Kucka, Ivana Bjelobaba, Melanija Tomić, Stanko S. Stojilkovic

**Affiliations:** ^1^Section on Cellular Signaling, Program in Developmental Neuroscience, Eunice Kennedy Shriver National Institute of Child Health and Human Development, National Institutes of Health, Bethesda, MD, USA

**Keywords:** lactotrophs, cAMP, cGMP, protein kinase A, calcium, prolactin, fusion pores

## Abstract

Lactotrophs are one of the five secretory anterior pituitary cell types specialized to synthesize and release prolactin. *In vitro*, these cells fire action potentials (APs) spontaneously and the accompanied Ca^2+^ transients are of sufficient amplitude to keep the exocytotic pathway, the transcription of prolactin gene, and *de novo* hormone synthesis continuously active. Basal cyclic nucleotide production is also substantial in cultured cells but not critical for the APs secretion/transcription coupling in lactotrophs. However, elevated intracellular cAMP levels enhance the excitability of lactotrophs by stimulating the depolarizing non-selective cationic hyperpolarization-activated cyclic nucleotide-regulated and background channels, whereas cGMP inhibits it by activating Ca^2+^-controlled K^+^ channels. Elevated cAMP also modulates prolactin release downstream of Ca^2+^ influx by changing the kinetic of secretory pores: stimulate at low and inhibit at high concentrations. Induction of prolactin gene and lactotroph proliferation is also stimulated by elevated cAMP through protein kinase A. Together, these observations suggest that in lactotrophs cAMP exhibits complex regulatory effects on voltage-gated Ca^2+^ influx and Ca^2+^-dependent cellular processes.

## Introduction

Lactotrophs are the anterior pituitary secretory cell type specialized to synthesize and release prolactin (PRL), a hormone with roles in reproduction, lactation, and metabolism. Depending on the sex and physiological status of the animals, lactotrophs comprise 20–50% of the cellular population of the anterior pituitary gland and, as somatotrophs, they are organized in large-scale networks (1–4). Ontogenetically, lactotrophs, together with somatotrophs and thyrotrophs, develop from the POU homeodomain transcriptional factor Pit-1-dependent lineage of the pituitary cells; Pit-1 is also necessary for the differentiation and proliferation of these cells. Specific regions responsible for Pit-1 transcriptional activity are found in the 5′ flanking region of PRL gene (*Prl*) and Pit-1-dependent *Prl* induction can be modified by different factors (5).

Lactotrophs are excitable cells and fire action potentials (APs) spontaneously; the accompanied Ca^2+^ influx is of sufficient amplitude to stimulate the exocytotic pathway by which prestored PRL in secretory vesicles is released (6). Voltage-gated Ca^2+^ influx also contributes to the control of other functions in pituitary cells, including the induction of *Prl* (7–9) and control of cell proliferation (10, 11). Such spontaneous electrical activity is silenced by numerous agonists, including dopamine, adenosine, endothelin-1, *gamma*-amino butyric acid (GABA), neuropeptide Y, and 5-HT, acting through G_i/o/z_ signaling pathway (12). In contrast, vasoactive intestinal peptide (VIP) facilitates voltage-gated Ca^2+^ influx through G_s_ signaling pathway (13). In mammals, Ca^2+^-mobilizing thyrotropin-releasing hormone (TRH), which comes from the hypothalamus, is a well-recognized PRL-releasing factor. Several other hypothalamic and intrapituitary agonists, including oxytocin, vasopressin, angiotensin II, acetylcholine, ATP, serotonin, and substance P, also stimulate PRL synthesis and release by facilitating Ca^2+^ mobilization from intracellular stores (14).

It is now well established that cyclic nucleotides also play important roles in lactotroph functions, a subject of this review. We will first briefly discuss cyclic nucleotide signaling pathways in pituitary cells: adenylyl cyclases (ACs), a family of enzymes that synthesize cAMP from ATP; guanylyl cyclases (GCs), which are responsible for production of cGMP from GTP; and phosphodiesterases (PDEs) and cyclic nucleotide transporters, accounting for control of intracellular cyclic nucleotide levels. This will be followed by review of data on the role of cyclic nucleotides in electrical activity, Ca^2+^ signaling, *Prl* expression, PRL synthesis and secretion, and cell proliferation. We will discuss both direct and indirect effects of cAMP and cGMP, mediated by cAMP-dependent kinase (PKA) and cGMP-dependent kinase (PKG). For the review of receptors modulating Ca^2+^ and cyclic nucleotide signaling in lactotrophs see Stojilkovic et al. (12). For interactions between intracellular calcium and cAMP in neuroendocrine cells see Antoni (15, 16).

## Pituitary Cyclic Nucleotides

**Figure 1:**
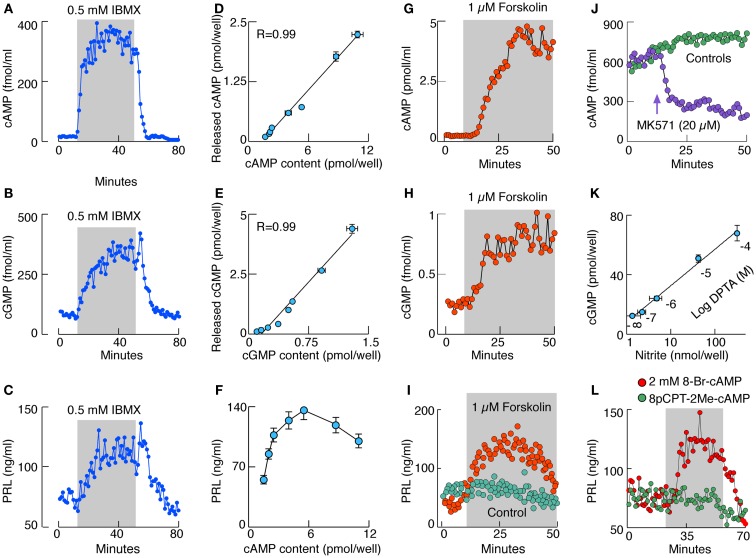
**Characterization of cyclic nucleotide signaling pathways in rat anterior pituitary cells *in vitro***. **(A–C)** Effect of IBMX, a PDEs inhibitor, on cAMP **(A)**, cGMP **(B)**, and PRL **(C)** release by perifused pituitary cells. **(D,E)** Correlation between released and cell content cAMP **(D)** and cGMP **(E)** in cells in static cultures treated with variable concentrations of IBMX. R, coefficient of correlation. **(F)** The relationship between cAMP intracellular content and PRL release. **(G–I)** Effects of forskolin, an AC activator, on cAMP **(G)**, cGMP **(H)**, and PRL **(I)** release by perifused pituitary cells. **(J)** Effect of MK571, an inhibitor of MRP transporters, on cAMP release in perifused pituitary cells. **(K)** Correlation between NO levels and cGMP production in cells stimulated with increasing doses of DPTA, an NO donor. **(L)** Stimulation of basal PRL release with 8-Br-cAMP but not with 8-pCPT-2Me-cAMP, an Epac cAMP receptor agonist. Gray areas indicate the duration of application of drugs.

The intracellular and extracellular cyclic nucleotide concentration in pituitary cells reflects the status of cAMP and cGMP *de novo* production and metabolism, the latter mediated by PDEs, a large family of enzymes, some specific for cAMP or cGMP and others less selective (3, 4). Pituitary cells express several PDEs (17, 18). In perifused pituitary cells, 0.5 mM 3-isobutyl-1-methylxantine (IBMX), a concentration that inhibits the majority of PDEs (19), increases the release of both cyclic nucleotides, reaching the steady-state levels within 10–15 min of application (Figures 1A,B). In cultured cells, IBMX induces a dose-dependent increase in cyclic nucleotide release and intracellular cAMP (Figure 1D) and cGMP (Figure 1E) accumulation. Although in both cases there is a linear relationship between intra and extracellular cyclic nucleotide levels, there is an obvious difference in the level of cAMP and cGMP intracellular accumulation; most of cGMP and only a fraction of cAMP is released.

These simple experiments show that there is a substantial basal cyclic nucleotide production in pituitary cells and that the intracellular cyclic nucleotide concentration is not only controlled by PDEs but also by the cyclic nucleotide export pump (20). This pump has a preference for cGMP, which in turn limits the intracellular cGMP accumulation and may represent an efficient way for the pituitary cells to limit cGMP/PKG-dependent intracellular signaling functions. Four lines of evidence indicate that multidrug resistance proteins MRP4 and MRP5 account for cyclic nucleotide transport in pituitary cells (21, 22): (i) the mRNA transcripts for MRP4 and MRP5 are present in pituitary cells. (ii) The MRP5 protein is also present in pituitary cells. (iii) Down-regulation of MRP5 expression in pituitary cells causes significant inhibition of cyclic nucleotide transport. (iv) MK571, a relatively specific inhibitor of MRPs, inhibits cyclic nucleotide transport in pituitary cells (Figure 1J).

In perifused pituitary cells there is a substantial basal PRL release, which further increases with IBMX-induced elevation in cyclic nucleotide levels, and returns to basal levels after its washout (Figure 1C). Such increase in PRL release is also observed when *de novo* protein synthesis is blocked, indicating that cyclic nucleotides affect the kinetics of basal PRL release from prestored secretory vesicles. A comparison of intracellular cAMP levels and PRL release suggests the bidirectional relationship: an increase in PRL release at lower cAMP levels and a decrease at higher cAMP levels (Figure 1F). In contrast, concentrations of intracellular cGMP reached during IBMX treatment do not affect basal PRL release (23).

Pituitary cells express several subtypes of ACs (24), including the Ca^2+^-inhibitable forms (23), but investigations of the cell type-specific expression of these enzymes have not been done. Figure 1A shows that pituitary ACs exhibit considerable basal activity. This is further confirmed by the inhibition of basal cAMP production with AC inhibitors (23). In lactotrophs, basal AC activity is inhibited by G_i/o/z_-coupled receptors activated by adenosine, dopamine, endothelin, GABA, neuropeptide Y, and 5-HT receptors, and is facilitated by the G_s_-coupled VIP/pituitary adenylate cyclase-activating peptide (PACAP) receptors (12). Both cAMP and cGMP release in perifused pituitary cells are stimulated by forskolin, a common activator of AC1–AC8 (Figures 1G, H). Similarly to IBMX, forskolin alone induces PRL release in a time-dependent manner (Figure 1I).

Pituitary cells also express the particulate (25, 26) and soluble GCs (27) responsible for synthesis of cGMP. It appears that the α1β1 soluble GC dimer is expressed in the anterior pituitary cells and accounts for the nitric oxide (NO)-dependent facilitation of cGMP production. Lactotrophs express NO synthases (NOS), a family of enzymes responsible for generation of NO and activation of soluble GC. Two calcium-sensitive NOS, endothelial and neuronal, are found in pituitary tissue and mixed cultured cells, as well as in enriched lactotroph and somatotroph fractions. Calcium-independent inducible NOS are also expressed in cultured lactotrophs and somatotrophs but only transiently, probably reflecting the influence of mechanical and enzymatic factors during dispersion of cells from intact tissue on the status of these enzymes (27). The G protein-coupled receptors contribute to the regulation of soluble GC activity in a Ca^2+^-dependent and -independent manner (28); the Ca^2+^-independent stimulation of the enzyme activity by G_s_-coupled receptors is mediated by PKA-dependent phosphorylation of the α subunit (29). This explains effects of forskolin on cGMP release (Figure 1H).

The NO dependence of cGMP production in pituitary cells is shown in experiments with DPTA, an NO donor (Figure 1K) (29). The activation of soluble GC is accompanied with inhibition rather than stimulation of PRL release in cultured pituitary cells, largely reflecting the cGMP-independent effects of NO on PRL release through a mechanism still not characterized (30). Application of 8-Br-cGMP, a cell-permeable cGMP analog, is unable to modulate PRL release. However, application of 8-Br-cAMP, a cell-permeable cAMP analog, mimics the effects of IBMX, and forskolin on PRL release (23), clearly indicating that elevation in cAMP but not cGMP levels facilitates the exocytotic pathways in these cells. This action is not mediated by Epac cAMP receptor, as application of 8pCPT-2Me-cAMP does not mimic the effects of 8-Br-cAMP on PRL release (Figure 1L).

## Roles of Cyclic Nucleotides in Excitability of Lactotrophs

**Figure 2:**
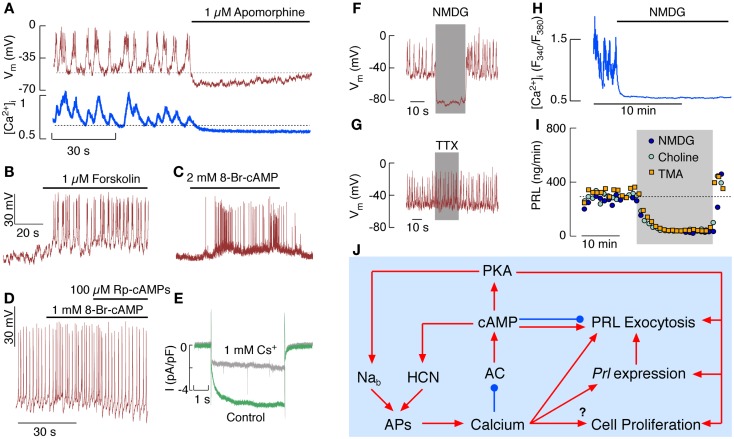
**Electrical activity and Ca^2+^ signaling in pituitary lactotrophs *in vitro***. **(A)** Simultaneous measurements of effects of apomorphine, a D2 receptor agonist, on spontaneous firing of APs (*top*) and Ca^2+^ signaling (*bottom*). **(B,C)** Effects of forskolin, an activator of ACs **(B)**, and 8-Br-cAMP, a cell-permeable cAMP analog **(C)**, on electrical activity of lactotrophs. **(D)** Effects of 8-Br-cAMP and Rp-cAMPs, a PKA inhibitor, on the frequency of APs in spontaneously firing lactotrophs. **(E)** Whole-cell voltage-clamp recording of I_h_ in the presence (gray) and absence (green) of 1 mM Cs^+^, an inhibitor of HCN channels. **(F,G)** Effects of complete replacement of bath Na^+^ with NMDG **(F)** and addition of tetrodotoxin (TTX) **(G)** on electrical activity in lactotrophs. **(H)** Effects of complete replacement of bath Na^+^ with NMDG on spontaneous Ca^2+^ signaling in lactotrophs. **(I)** Effects of complete replacement of bath Na^+^ with NMDG, choline chloride, and TMA on basal PRL release. **(J)** Schematic representation of effects of intracellular Ca^2+^ and cAMP on lactotroph function.

*In vitro*, isolated lactotrophs generate APs independently of external stimuli, a phenomenon termed spontaneous electrical activity. Both types of electrical activity, single spiking and plateau bursting, are observed (12); the latter type being generator of global Ca^2+^ transients (Figure 2A). The physiological significance of such spontaneous electrical activity in lactotrophs is now well established. The cells *in vitro* release PRL in the absence of external stimuli termed basal or spontaneous release (14). High PRL release is also observed in animals bearing ectopic pituitary grafts (31). In both cases, spontaneous APs and the associated Ca^2+^ influx account for high steady-state PRL release and any maneuver leading to silencing of electrical activity also abolishes Ca^2+^ influx and basal PRL release (32). Continuous PRL release does not affect the intracellular PRL pool significantly in perifused pituitary cells, because basal *Prl* expression is sufficient to keep *de novo* hormone synthesis (33) in part due to role of Ca^2+^ in induction of this gene (9).

Spontaneous firing of APs and basal PRL secretion are not critically dependent on the status of intracellular cyclic nucleotides; inhibition of basal AC activity decreases but does not abolish basal PRL release and has no effect on spontaneous firing of APs. Inhibition of NOS and soluble GC is also ineffective, as well as inhibition of PKA and PKG (23). Thus, spontaneous firing of APs is an intrinsic feature of lactotrophs and, at least in a fraction of lactotrophs, firing of AP accounts for basal PRL release.

The hypothalamic dopamine, acting through dopamine D_2_ receptors, is the main physiological inhibitor of spontaneous electrical activity and the coupled synthesis and release of PRL (34). Figure 2A shows a rapid effect of a D2 receptor agonist on spontaneous electrical activity and voltage-gated Ca^2+^ influx. Both dopamine and endothelin-1 also inhibit firing of AP and PRL release in cells with elevated cAMP, indicating that these agonists utilize other pathways to silence lactotrophs (33, 35). These include activation of inwardly rectifying K^+^ channels and inhibition of voltage-gated Ca^2+^ channels, causing abolition of electrical activity and basal PRL secretion independently of the status of AC activity (33, 34).

However, elevation of intracellular cAMP levels stimulates electrical activity in quiescent cells and increases the AP frequency in spontaneously firing cells (23, 36); Figure 2B illustrates effects of forskolin and Figure 2C effects of 8-Br-cAMP on initiation of firing of AP, whereas Figure 2D shows increase in the frequency of spiking in a spontaneously active cell. Such stimulation occurs both in a PKA-independent and -dependent manner. The PKA-independent facilitation of electrical activity probably involves direct effects of cAMP on hyperpolarization-activated cyclic nucleotide-regulated (HCN) channels, which are expressed in GH_3_ lacto-somatotrophs and in a fraction of lactotrophs (22, 37, 38) and are inhibited by 1 mM Cs^+^ in bath medium (Figure 2E).

Figure 2D shows the PKA-dependent facilitation of electrical activity; inhibition of PKA by Rp-cAMPs eliminates an increase in the firing frequency generated by 8-Br-AMP, but does not abolish spontaneous electrical activity. The channels responsible for PKA-dependent facilitation of electrical activity in lactotrophs have not been identified, but several recent findings indicate that phosphorylation of a putative Na^+^-dependent background (Na_b_) channels may account for it. The expression of this channel in lactotrophs and immortalized GH_3_ lacto-somatotrophs was originally proposed by Simasko’s group (39, 40) using a protocol shown in Figures 2F–I. Replacement of extracellular Na^+^ with NMDG, a large organic cation, causes an instantaneous hyperpolarization of cell membrane and abolition of spontaneous AP firing (Figure 2F), indicating the dependence of the baseline membrane potential on Na_b_ conductance. Voltage-gated Na^+^ channels do not account for this conductance, because tetrodotoxin, a specific blocker of these channels, does not abolish spontaneous firing of APs (Figure 2G). In intact cells, replacement of bath Na^+^ with NMDG^+^ abolishes spontaneous Ca^2+^ spiking (Figure 2H). Basal PRL release is also abolished by replacement of bath Na^+^ with NMDG^+^ and other large cations (Figure 2I). The Na_b_ conductance was also reduced in lactotrophs with various inhibitors of cation channels, including TRP channels, causing cessation of electrical activity and inhibition of PRL release (41). This could indicate that a member of TRP family of channels accounts for the Na_b_ conductance in lactotrophs and other secretory pituitary cell types (42).

Numerous experiments performed with normal rat and mouse pituitary cells, as well as in pituitary cells haploinsufficient for the main PKA catalytic subunit, are consistent with a hypothesis that Na_b_ channels are stimulated by PKA and contribute to Ca^2+^ signaling and basal PRL release indirectly, by controlling the pacemaking depolarization (42). It also appears that the MRP4/5-mediated cyclic nucleotide efflux can be rapidly modulated by membrane potential of anterior pituitary cells determined by Na_b_ conductance (22). At the present time, it is not clear what is the mechanism and physiological role of regulation of MRP4/5 activity by membrane potential.

In contrast to cAMP, cGMP is unlikely to play an important role in control of lactotroph functions. This could reflect the preference of MRP4/5 for cGMP, not allowing substantial intracellular accumulation of this second messenger. Still, it is possible that cGMP inhibits spontaneous excitability of lactotrophs by activating the BK-type of Ca^2+^-controlled K^+^ channels in a PKG-dependent manner (20). The presence of NOS signaling system in these cells may provide an additional and cGMP-independent mechanism for inhibition of PRL secretion (30).

## Effects of Camp on PRL Release Downstream of Ca^2+^ Influx

Both cAMP and its kinase also play important roles in the control of pituitary cell functions independently of Ca^2+^ signaling. This includes stimulation of the exocytotic pathway downstream of the voltage-gated Ca^2+^ influx (43). Recently, we clarified the mechanism by which cAMP at high concentrations inhibits PRL release. By using high-resolution capacitance measurements, which represent elementary exocytotic events, increase in the frequency of transient exocytotic events was detected. Basically, cAMP mediated stabilization of wide fusion pores prevents secretory vesicles in lactotrophs from proceeding to the full-fusion stage of exocytosis, which hinders vesicle content discharge at high cAMP concentrations (44). Further investigations are needed to separate direct effects of cAMP from those mediated by PKA on the exocytotic pathway in lactotrophs downstream of Ca^2+^ influx.

## Other Roles of Cyclic Nucleotides in Lactotrophs

*Prl* transcription is regulated by cAMP through PKA signaling pathway (45) in a cAMP response element-binding protein-independent manner (46). It has also been shown that G_s_ alpha subunits stimulate Pit-1 promoter via a PKA-mediated signaling pathway (47). PACAP also facilitates *Prl* expression in immortalized lacto-somatotrophs in a PKA-dependent manner, in contrast to TRH, which facilitates transcription in a Ca^2+^-dependent manner (48). Furthermore, there is a PKA-dependent but Pit-1-independent effect of cAMP on *Prl* expression (49).

cAMP and PKA contribute to the control of lactotroph proliferation. Down-regulation of basal AC by dopamine is known to inhibit not only *Prl* expression but also lactotroph proliferation (50). It appears that the cAMP-dependent cell proliferation requires the MAPK signaling pathway (51). It has been suggested that the cAMP response element-binding protein is involved in the regulation of cell proliferation and *Prl* expression in normal lactotrophs (52). VIP also facilitates lactotroph proliferation through MAPK signaling pathway (53).

## Summary

The interactions between Ca^2+^ and cAMP in control of lactotroph functions are summarized in Figure 2J. Spontaneous electrical activity and AP secretion coupling are sufficient to keep lactotrophs operative in the absence of any stimuli due to refilling of the PRL secretory pool by Ca^2+^-dependent *Prl* expression and *de novo* protein synthesis. AP-driven Ca^2+^ influx controls basal cAMP levels by inhibiting AC5/6 isoforms and stimulating PDEs. Still the residual cAMP levels are sufficient to integrate HCN channels in spontaneous electrical activity and probably to facilitate exocytosis downstream of voltage-gated Ca^2+^ influx. Ca^2+^ signaling function in lactotrophs is facilitated by numerous Ca^2+^-mobilizing receptors expressed in these cells, and their activation transiently inhibits electrical activity, followed by a sustained increase in the firing frequency. Detailed studies are needed to clarify whether and how many of these receptors cross-couple to the G_s_ signaling pathway. Experiments with IBMX, forskolin, and cell-permeable cAMP analogs show that cAMP directly stimulates HCN channels and through PKA stimulates still unidentified Na_b_ channels, and probably influences gating of several other channels. The cAMP-dependent modulation of PRL release reflects the effects of this messenger on excitability of lactotrophs, the accompanied voltage-gated Ca^2+^ influx and Ca^2+^-dependent exocytosis of the prestored PRL, as well as its influence on the exocytotic pathway downstream of voltage-gated Ca^2+^ influx. cAMP exhibits bidirectional effects on the kinetics of secretory pores, by facilitating it at lower concentrations and stabilizing it at higher concentrations. Further studies are needed to clarify to which extent cAMP, PKA, or Epac cAMP receptors contribute to this process. The AC-signaling pathway provides a very powerful pathway for stimulation of electrical activity, cell proliferation, *Prl* expression, and exocytosis. However, only VIP and PACAP use this pathway to stimulate lactotroph function by activating VPAC_2_ receptors coupled to the G_s_ signaling pathway. In contrast, at least six G_i/o/z_-coupled receptors activated by adenosine, dopamine, endothelin-1, GABA, neuropeptide Y, and 5-HT, silence both AC activity and spontaneous electrical activity, clearly indicating that the main role of hypothalamic and intrapituitary regulation is to slow down this runaway engine.

## Conflict of Interest Statement

The authors declare that the research was conducted in the absence of any commercial or financial relationships that could be construed as a potential conflict of interest.

## References

[1] Bonnefont X Lacampagne A Sanchez-Hormigo A Fino E Creff A Mathieu MN Revealing the large-scale network organization of growth hormone-secreting cells. Proc Natl Acad Sci U S A (2005) 102:16880–5 10.1073/pnas.0508202102 16272219 PMC1277257

[2] Schaeffer M Hodson DJ Lafont C Mollard P Endocrine cells and blood vessels work in tandem to generate hormone pulses. J Mol Endocrinol (2011) 47:R59–66 10.1530/JME-11-0035 21622530

[3] Hodson DJ Romano N Schaeffer M Fontanaud P Lafont C Fiordelisio T Coordination of calcium signals by pituitary endocrine cells in situ. Cell Calcium (2012) 51:222–30 10.1016/j.ceca.2011.11.007 22172406

[4] Hodson DJ Schaeffer M Romano N Fontanaud P Lafont C Birkenstock J Existence of long-lasting experience-dependent plasticity in endocrine cell networks. Nat Commun (2012) 3:605 10.1038/ncomms1612 22215080 PMC3272579

[5] Quentien MH Barlier A Franc JL Pellegrini I Brue T Enjalbert A Pituitary transcription factors: from congenital deficiencies to gene therapy. J Neuroendocrinol (2006) 18:633–42 10.1111/j.1365-2826.2006.01461.x 16879162

[6] Stojilkovic SS Murano T Gonzalez-Iglesias AE Andric SA Popovic MA Van Goor F Multiple roles of Gi/o protein-coupled receptors in control of action potential secretion coupling in pituitary lactotrophs. Ann N Y Acad Sci (2009) 1152:174–86 10.1111/j.1749-6632.2008.03994.x 19161388 PMC2733166

[7] White BA Bauerle LR Bancroft FC Calcium specifically stimulates prolactin synthesis and messenger RNA sequences in GH3 cells. J Biol Chem (1981) 256:5942–5 7240183

[8] Jackson AE Bancroft C Proximal upstream flanking sequences direct calcium regulation of the rat prolactin gene. Mol Endocrinol (1988) 2:1139–44 10.1210/mend-2-11-1139 2464750

[9] Hoggard N Davis JR Berwaer M Monget P Peers B Belayew A Pit-1 binding sequences permit calcium regulation of human prolactin gene expression. Mol Endocrinol (1991) 5:1748–54 10.1210/mend-5-11-1748 1779976

[10] Ramsdell JS Voltage-dependent calcium channels regulate GH4 pituitary cell proliferation at two stages of the cell cycle. J Cell Physiol (1991) 146:197–206 10.1002/jcp.1041460203 1705563

[11] Van Dolah FM Ramsdell JS Maitotoxin, a calcium channel activator, inhibits cell cycle progression through the G1/S and G2/M transitions and prevents CDC2 kinase activation in GH4C1 cells. J Cell Physiol (1996) 166:49–56 10.1002/(SICI)1097-4652(199601)166:1<49::AID-JCP6>3.0.CO;2-G 8557775

[12] Stojilkovic SS Tabak J Bertram R Ion channels and signaling in the pituitary gland. Endocr Rev (2010) 31:845–915 10.1210/er.2010-0005 20650859 PMC3365841

[13] Al Kahtane A Kannan M Kang SW El Halawani ME Regulation of prolactin gene expression by vasoactive intestinal peptide and dopamine in the turkey: role of Ca signalling. J Neuroendocrinol (2005) 17:649–55 10.1111/j.1365-2826.2005.01352.x 16159377

[14] Freeman ME Kanyicska B Lerant A Nagy G Prolactin: structure, function, and regulation of secretion. Physiol Rev (2000) 80:1523–631 11015620 10.1152/physrev.2000.80.4.1523

[15] Antoni FA Interactions between intracellular free Ca^2^+ and cyclic AMP in neuroendocrine cells. Cell Calcium (2012) 51:260–6 10.1016/j.ceca.2011.12.013 22385836

[16] Antoni FA New paradigms in cAMP signalling. Mol Cell Endocrinol (2012) 353:3–9 10.1016/j.0.e.2011.10.034 22085559

[17] Persani L Borgato S Lania A Filopanti M Mantovani G Conti M Relevant cAMP-specific phosphodiesterase isoforms in human pituitary: effect of Gs(alpha) mutations. J Clin Endocrinol Metab (2001) 86:3795–800 10.1210/jc.86.8.3795 11502813

[18] Ang KL Antoni FA Functional plasticity of cyclic AMP hydrolysis in rat adenohypophysial corticotroph cells. Cell Signal (2002) 14:445–52 10.1016/S0898-6568(01)00267-4 11882389

[19] Bender AT Beavo JA Cyclic nucleotide phosphodiesterases: molecular regulation to clinical use. Pharmacol Rev (2006) 58:488–520 10.1124/pr.58.3.5 16968949

[20] Stojilkovic SS Kretschmannova K Tomic M Stratakis CA Dependence of the excitability of pituitary cells on cyclic nucleotides. J Neuroendocrinol (2012) 24:1183–200 10.1111/j.1365-2826.2012.02335.x 22564128 PMC3421050

[21] Andric SA Kostic TS Stojilkovic SS Contribution of multidrug resistance protein MRP5 in control of cyclic guanosine 5′-monophosphate intracellular signaling in anterior pituitary cells. Endocrinology (2006) 147:3435–45 10.1210/en.2006-0091 16614078

[22] Kucka M Kretschmannova K Murano T Wu CP Zemkova H Ambudkar SV Dependence of multidrug resistance protein-mediated cyclic nucleotide efflux on the background sodium conductance. Mol Pharmacol (2010) 77:270–9 10.1124/mol.109.059386 19903828 PMC2812068

[23] Gonzalez-Iglesias AE Jiang Y Tomic M Kretschmannova K Andric SA Zemkova H Dependence of electrical activity and calcium influx-controlled prolactin release on adenylyl cyclase signaling pathway in pituitary lactotrophs. Mol Endocrinol (2006) 20:2231–46 10.1210/me.2005-0363 16645040

[24] Antoni FA Sosunov AA Haunso A Paterson JM Simpson J Short-term plasticity of cyclic adenosine 3′,5′-monophosphate signaling in anterior pituitary corticotrope cells: the role of adenylyl cyclase isotypes. Mol Endocrinol (2003) 17:692–703 10.1210/me.2002-0369 12554775

[25] McArdle CA Olcese J Schmidt C Poch A Kratzmeier M Middendorff R C-type natriuretic peptide (CNP) in the pituitary: is CNP an autocrine regulator of gonadotropes? Endocrinology (1994) 135:2794–801 10.1210/en.135.6.2794 7988473

[26] Grandclement B Brisson C Bayard F Tremblay J Gossard F Morel G Localization of mRNA coding for the three subtypes of atrial natriuretic factor (ANF) receptors in rat anterior pituitary gland cells. J Neuroendocrinol (1995) 7:939–48 10.1111/j.1365-2826.1995.tb00739.x 8745272

[27] Kostic TS Andric SA Stojilkovic SS Spontaneous and receptor-controlled soluble guanylyl cyclase activity in anterior pituitary cells. Mol Endocrinol (2001) 15:1010–22 10.1210/me.15.6.1010 11376118

[28] Kostic TS Tomic M Andric SA Stojilkovic SS Calcium-independent and cAMP-dependent modulation of soluble guanylyl cyclase activity by G protein-coupled receptors in pituitary cells. J Biol Chem (2002) 277:16412–8 10.1074/jbc.M112439200 11867632

[29] Kostic TS Andric SA Stojilkovic SS Receptor-controlled phosphorylation of alpha 1 soluble guanylyl cyclase enhances nitric oxide-dependent cyclic guanosine 5′-monophosphate production in pituitary cells. Mol Endocrinol (2004) 18:458–70 10.1210/me.2003-0015 14630997

[30] Andric SA Gonzalez-Iglesias AE Van Goor F Tomic M Stojilkovic SS Nitric oxide inhibits prolactin secretion in pituitary cells downstream of voltage-gated calcium influx. Endocrinology (2003) 144:2912–21 10.1210/en.2002-0147 12810546

[31] Maric D Simonovic I Kovacevic R Krsmanovic L Stojilkovic S Andjus RK Effects of short-term and long-term hyperprolactinemia on the developmental pattern of androgen and LH levels in the immature male rat. J Endocrinol Invest (1982) 5:235–41 7175107 10.1007/BF03348329

[32] Van Goor F Zivadinovic D Martinez-Fuentes AJ Stojilkovic SS Dependence of pituitary hormone secretion on the pattern of spontaneous voltage-gated calcium influx. Cell type-specific action potential secretion coupling. J Biol Chem (2001) 276:33840–6 11457854 10.1074/jbc.M105386200

[33] Gonzalez-Iglesias AE Murano T Li S Tomic M Stojilkovic SS Dopamine inhibits basal prolactin release in pituitary lactotrophs through pertussis toxin-sensitive and -insensitive signaling pathways. Endocrinology (2008) 149:1470–9 10.1210/en.2007-0980 18096663 PMC2276716

[34] Missale C Nash SR Robinson SW Jaber M Caron MG Dopamine receptors: from structure to function. Physiol Rev (1998) 78:189–225 9457173 10.1152/physrev.1998.78.1.189

[35] Andric SA Zivadinovic D Gonzalez-Iglesias AE Lachowicz A Tomic M Stojilkovic SS Endothelin-induced, long lasting, and Ca^2^+ influx-independent blockade of intrinsic secretion in pituitary cells by Gz subunits. J Biol Chem (2005) 280:26896–903 10.1074/jbc.M502226200 15919662

[36] Kretschmannova K Kucka M Gonzalez-Iglesias AE Stojilkovic SS The expression and role of hyperpolarization-activated and cyclic nucleotide-gated channels in endocrine anterior pituitary cells. Mol Endocrinol (2012) 26:153–64 10.1210/me.2011-1207 22135067 PMC3248322

[37] Gonzalez-Iglesias AE Kretschmannova K Tomic M Stojilkovic SS ZD7288 inhibits exocytosis in an HCN-independent manner and downstream of voltage-gated calcium influx in pituitary lactotrophs. Biochem Biophys Res Commun (2006) 346:845–50 10.1016/j.bbrc.2006.05.194 16780797

[38] Kretschmannova K Gonzalez-Iglesias AE Tomic M Stojilkovic SS Dependence of hyperpolarisation-activated cyclic nucleotide-gated channel activity on basal cyclic adenosine monophosphate production in spontaneously firing GH3 cells. J Neuroendocrinol (2006) 18:484–93 10.1111/j.1365-2826.2006.01438.x 16774497

[39] Simasko SM A background sodium conductance is necessary for spontaneous depolarizations in rat pituitary cell line GH3. Am J Physiol (1994) 266:C709–19 8166234 10.1152/ajpcell.1994.266.3.C709

[40] Sankaranarayanan S Simasko SM A role for a background sodium current in spontaneous action potentials and secretion from rat lactotrophs. Am J Physiol (1996) 271:C1927–34 8997194 10.1152/ajpcell.1996.271.6.C1927

[41] Kucka M Kretschmannova K Stojilkovic SS Zemkova H Tomic M Dependence of spontaneous electrical activity and basal prolactin release on nonselective cation channels in pituitary lactotrophs. Physiol Res (2012) 61:267–75 22480423 10.33549/physiolres.932301 PMC3674129

[42] Tomic M Kucka M Kretschmannova K Li S Nesterova M Stratakis CA Role of nonselective cation channels in spontaneous and protein kinase A-stimulated calcium signaling in pituitary cells. Am J Physiol Endocrinol Metab (2011) 301:E370–9 10.1152/ajpendo.00130.2011 21586701 PMC3154538

[43] Sikdar SK Zorec R Mason WT cAMP directly facilitates Ca-induced exocytosis in bovine lactotrophs. FEBS Lett (1990) 273:150–4 10.1016/0014-5793(90)81072-V 2172025

[44] Calejo AI Jorgacevski J Kucka M Kreft M Goncalves PP Stojilkovic SS cAMP-mediated stabilization of fusion pores in cultured rat pituitary lactotrophs. J Neurosci (2013) 33:8068–78 10.1523/JNEUROSCI.5351-12.2013 23637196 PMC3674111

[45] Diamond SE Chiono M Gutierrez-Hartmann A Reconstitution of the protein kinase A response of the rat prolactin promoter: differential effects of distinct Pit-1 isoforms and functional interaction with Oct-1. Mol Endocrinol (1999) 13:228–38 10.1210/me.13.2.228 9973253

[46] Zanger K Cohen LE Hashimoto K Radovick S Wondisford FE A novel mechanism for cyclic adenosine 3′,5′-monophosphate regulation of gene expression by CREB-binding protein. Mol Endocrinol (1999) 13:268–75 10.1210/me.13.2.268 9973256

[47] Gaiddon C Tian J Loeffler JP Bancroft C Constitutively active G(S) alpha-subunits stimulate Pit-1 promoter activity via a protein kinase A-mediated pathway acting through deoxyribonucleic acid binding sites both for Pit-1 and for adenosine 3′,5′-monophosphate response element-binding protein. Endocrinology (1996) 137:1286–91 10.1210/en.137.4.1286 8625901

[48] Coleman DT Chen X Sassaroli M Bancroft C Pituitary adenylate cyclase-activating polypeptide regulates prolactin promoter activity via a protein kinase A-mediated pathway that is independent of the transcriptional pathway employed by thyrotropin-releasing hormone. Endocrinology (1996) 137:1276–85 10.1210/en.137.4.1276 8625900

[49] Gellersen B Kempf R Telgmann R Dimattia GE Pituitary-type transcription of the human prolactin gene in the absence of Pit-1. Mol Endocrinol (1995) 9:887–901 10.1210/me.9.7.887 7476971

[50] Ben-Jonathan N Hnasko R Dopamine as a prolactin (PRL) inhibitor. Endocr Rev (2001) 22:724–63 10.1210/er.22.6.724 11739329

[51] Suzuki S Yamamoto I Arita J Mitogen-activated protein kinase-dependent stimulation of proliferation of rat lactotrophs in culture by 3′,5′-cyclic adenosine monophosphate. Endocrinology (1999) 140:2850–8 10.1210/en.140.6.2850 10342877

[52] Ishida M Mitsui T Yamakawa K Sugiyama N Takahashi W Shimura H Involvement of cAMP response element-binding protein in the regulation of cell proliferation and the prolactin promoter of lactotrophs in primary culture. Am J Physiol Endocrinol Metab (2007) 293:E1529–37 10.1152/ajpendo.00028.2007 17925456

[53] Fernandez M Sanchez-Franco F Palacios N Sanchez I Cacicedo L IGF-I and vasoactive intestinal peptide (VIP) regulate cAMP-response element-binding protein (CREB)-dependent transcription via the mitogen-activated protein kinase (MAPK) pathway in pituitary cells: requirement of Rap1. J Mol Endocrinol (2005) 34:699–712 10.1677/jme.1.01703 15956341

